# Supplementary material for: Content-rich biological network constructed by mining PubMed abstracts
Source: BMC Bioinformatics. 2004 Oct 8;5:147. doi: 10.1186/1471-2105-5-147 (PMC528731; doi:10.1186/1471-2105-5-147)
Supplement: Additional File 2 — The original results of the above study (non-essential files are deleted to keep the file size under the limit set by BMC bioinformatics). [file 1471-2105-5-147-S2.bz2 › chilibotAdditionalFile2/dip20/348ID7721786E4496/html/IL4R_IL13.html]

 


 **IL4R** and **IL13** 
  
Found 306 abstracts in PubMed, retrieved 20.  
 

 What does Google say? 
 PDF only 
| .edu only 

---

**Interactive relationship** (e.g. stimulation, inhibition, etc)

**Neutral relationship**- Association and Interaction of the  **IL4R** , IL4, and  **IL13**  Loci with Type 1 Diabetes among Filipinos.  Ref: 12748907 Am J Hum Genet, 2003

**Non-interactive relationship** (e.g. studied together, co-existance, homology, etc.)

- The most recent experimental observations suggest that a critical role in the modulation of immune response is sustained by Th2 cytokines, such as interleukin 4  [ **IL4R** ]  IL 4, IL 5 and IL 13  [ **IL13** ] , by co stimulatory molecules, such as CD40 on B cells, and by hormones and neuropeptides.  Ref: 12787302 Pediatr Allergy Immunol, 2003
- Histamine enhances the secretion of Th2 cytokines such as IL 4 interleukin 4  [ **IL4R** ] , IL 5, IL 10 and IL 13  [ **IL13** ]  and inhibits the production of Th1 cytokines IL 2 and IFNgamma interferon gamma and monokine IL 12.  Ref: 12810348 Int Immunopharmacol, 2003
- Interleukin 4  [ **IL4R** ]  and interleukin 13  [ **IL13** ]  signaling connections maps.  Ref: 12791978 Science, 2003
- Since IL4 and  **IL13**  both serve as ligands for a receptor composed, in part, of the  **IL4R**  alpha chain, we looked for potential epistasis between polymorphisms in the  **IL4R**  locus on chromosome 16p11 and the five SNPs in the IL4 and  **IL13**  loci on chromosome 5q31 and found, through use of a logistic regression model, significant gene gene interactions P=.045, corrected for multiple comparisons by permutation analysis.  Ref: 12748907 Am J Hum Genet, 2003
- BACKGROUND Signal transducer and activator of transcription 6 STAT6 is a key transcription factor involved in both interleukin 4  [ **IL4R** ]  IL 4 and IL 13  [ **IL13** ]  mediated biological responses, such as allergies.  Ref: 12759487 Int Arch Allergy Immunol, 2003
- Our data suggest that the risk for T1D is determined, in part, by polymorphisms within the  **IL4R**  locus, including promoter and coding sequence variants, and by specific combinations of genotypes at the  **IL4R**  and the IL4 and  **IL13**  loci.  Ref: 12748907 Am J Hum Genet, 2003
- Interleukin 4  [ **IL4R** ]  IL 4 and IL 13  [ **IL13** ]  are two cytokines produced by T helper type 2 cells, mast cells, and basophils.  Ref: 12791978 Science, 2003
- We determined the allele frequencies of five polymorphisms in the  **IL13** , IL13RA1, and IL4RA  [ **IL4R** ]  genes in 588 continuing smokers chosen from the NHLBI Lung Health Study for having the fastest n = 282 and slowest n = 306 5 yr rate of decline of lung function mean change in FEV 1 %predicted yr = 4.1 and 1.1, respectively.  Ref: 12594065 Am J Respir Cell Mol Biol, 2003
- Polymorphisms in the  **IL13** , IL13RA1, and IL4RA  [ **IL4R** ]  genes and rate of decline in lung function in smokers.  Ref: 12594065 Am J Respir Cell Mol Biol, 2003
- Here we show that mice lacking interleukin 12p35 IL 12p35 following T. crassiceps infection, failed to mount a Th1 response, but developed a strong Th2 type response, produced higher levels of IgG1, IgE, interleukin 4  [ **IL4R** ] , interleukin 5 as well as interleukin 13  [ **IL13** ]  than wild type mice, and became highly susceptible to the larval stage of this cestode.  Ref: 12814650 Int J Parasitol< MedlineTA>Int J Parasitol, 2003
- Although there were no significant differences in the levels of interleukin 4  [ **IL4R** ] , IL 5, IL 13  [ **IL13** ] , eotaxin, granulocyte macrophage colony stimulating factor, RANTES, or eosinophil cationic protein, there was a marked increase in eosinophil derived neurotoxin both systemically and in the lungs of individuals with TPE compared to each of the control groups.  Ref: 12595450 Infect Immun, 2003
- Among the cytokines inhibited were interleukin 4  [ **IL4R** ]  IL 4, IL 13  [ **IL13** ] , IFNgamma, tumour necrosis factor alpha TNFalpha and granulocyte macrophage colony stimulating factor.  Ref: 12777636 Rheumatology Oxford < MedlineTA>Rheumatology Oxford , 2003
- Using an in vitro cell culture system, we investigated how the key regulatory cytokines interleukin 4  [ **IL4R** ]  IL 4, IL 10, IL 13  [ **IL13** ] , and TGF beta might modulate the effect of IFN gamma in inducing resistance to infection in enterocyte cell lines.  Ref: 12874337 Infect Immun, 2003
- We hypothesized that variants in the  **IL13** , IL13RA1, and IL4RA  [ **IL4R** ]  genes would be associated with an accelerated rate of decline of lung function among smokers.  Ref: 12594065 Am J Respir Cell Mol Biol, 2003
- The roles of interleukin 4  [ **IL4R** ]  IL 4 and IL 13  [ **IL13** ]  in the regulation of immunity to Leishmania donovani infection are still poorly understood.  Ref: 12874364 Infect ImmunInfect Immun, 2003
- Signal transducer and activator of transcription 6 STAT6 is a key transcription factor involved in interleukin 4  [ **IL4R** ]  IL 4 and IL 13  [ **IL13** ]  mediated Th2 response.  Ref: 12605697 Clin Exp Immunol, 2003
- Neutralizing antibodies against human RANTES, MIP 1alpha, MIP 1beta, alpha interferon IFN alpha, IFN beta, IFN gamma, interleukin 4  [ **IL4R** ]  IL 4, IL 10, IL 13  [ **IL13** ] , IL 16, MCP 1, MCP 3, tumor necrosis factor alpha TNF alpha, or TNF beta failed to reverse the HIV 1 suppressive activity.  Ref: 12885891 J VirolJ Virol, 2003
- Both interleukin 4  [ **IL4R** ]  IL 4 and IL 13  [ **IL13** ]  inhibited TNF alpha and IFN gamma enhanced MDC production in HaCaT cells in a dose dependent manner.  Ref: 12670721 J Dermatol Sci, 2003
- Throughout the course of infection, Taenia crassiceps soluble antigen stimulated spleen cells from both MIF and MIF mice produced significant and comparable levels of interleukin 4  [ **IL4R** ]  IL 4, but those from MIF mice produced significantly more IL 13  [ **IL13** ] , as well as gamma interferon IFN gamma, suggesting that the susceptibility of MIF mice to T. crassiceps was not due to the lack of IFN gamma production.  Ref: 12595439 Infect Immun, 2003
- Haplotypes consisting of alleles from the  **IL13**  polymorphisms or from the IL4RA  [ **IL4R** ]  polymorphisms were not associated with rate of decline in lung function in smokers.  Ref: 12594065 Am J Respir Cell Mol Biol, 2003
- Furthermore, cysteine proteinases induced neither interleukin 4  [ **IL4R** ]  IL 4 nor IL 13  [ **IL13** ]  and low levels of IL 10 in controls and patients.  Ref: 12704171 Infect Immun, 2003
- In contrast, similarly infected CD40 deficient BALB c mice CD40 displayed impairment of both Th1 and Th2 type responses associated with low levels of interferon gamma as well as IgE, interleukin 4  [ **IL4R** ] , interleukin 5 and interleukin 13  [ **IL13** ] , but efficiently controlled T. crassiceps infection.  Ref: 12814650 Int J Parasitol< MedlineTA>Int J Parasitol, 2003
- Interleukin 4  [ **IL4R** ]  and IL 13  [ **IL13** ]  activated phosphorylation of mitogen activated protein kinase in LS174T cells.  Ref: 12848848 Immunol Cell Biol, 2003
- Ten single nucleotide polymorphisms SNPs, including two promoter SNPs in the  **IL4R**  locus on chromosome 16p11, one promoter SNP in the IL4 locus on chromosome 5q31, and four SNPs including two promoter SNPs in the  **IL13**  locus on chromosome 5q31 were examined for association, linkage disequilibrium, and interaction.  Ref: 12748907 Am J Hum Genet, 2003
